# Using the technology acceptance model to explore health provider and administrator perceptions of the usefulness and ease of using technology in palliative care

**DOI:** 10.1186/s12904-020-00644-8

**Published:** 2020-09-07

**Authors:** M. Nguyen, J. Fujioka, K. Wentlandt, N. Onabajo, I. Wong, R. S. Bhatia, O. Bhattacharyya, V. Stamenova

**Affiliations:** Women’s College Hospital Institute for Health System Solutions and Virtual Care, 76 Grenville Street, 6th Floor, Toronto, Ontaro M5S 1B2 Canada

**Keywords:** Palliative care, Health technology, Telehealth, End-of-life

## Abstract

**Background:**

Studies have shown that telehealth applications in palliative care are feasible, can improve quality of care, and reduce costs but few studies have focused on user acceptance of current technology applications in palliative care. Furthermore, the perspectives of health administrators have not been explored in palliative care and yet they are often heavily involved, alongside providers, in the coordination and use of health technologies. The study aim was to explore both health care provider and administrator perceptions regarding the usefulness and ease of using technology in palliative care.

**Methods:**

The Technology Acceptance Model (TAM) was used as the guiding theoretical framework to provide insight into two key determinants that influence user acceptance of technology (perceived usefulness and ease of use). Semi-structured interviews (*n* = 18) with health providers and administrators with experience coordinating or using technology in palliative care explored the usefulness of technologies in palliative care and recommendations to support adoption. Interview data were analyzed using inductive thematic analysis to identify common, meaningful themes.

**Results:**

Four themes were identified; themes related to perceived usefulness were: enabling remote connection and information-sharing platform. Themes surrounding ease of use included: integration with existing IT systems and user-friendly with ready access to technical support. Telehealth can enable remote connection between patients and providers to help address insufficiencies in the current palliative care environment. Telehealth, as an information sharing platform, could support the coordination and collaboration of interdisciplinary providers caring for patients with palliative needs. However, health technologies need to passively integrate with existing IT systems to enhance providers’ workflow and productivity. User-friendliness with ready access to technical support was considered especially important in palliative care as patients often experience diminished function.

**Conclusion:**

Participants’ perspectives of technology acceptance in palliative care were largely dependent on their potential to help address major challenges in the field without imposing significant burden on providers and patients.

## Background

Approximately 40 million people worldwide require palliative care each year, and yet, less than 15% of these individuals currently receive it [1]. The increasing aging population and growing prevalence of chronic conditions will continue to drive demand for palliative services [2, 3]. The COVID-19 pandemic has created a rapid surge in demand for remote palliative care services that will require rapid scale up [4] and minimize both patients’ and providers’ exposure to the virus. Considering the urgent need and likely long-lasting impacts of the pandemic, the need for remote palliative services will exist both in the short- and long-term. Outside of a pandemic, meeting the palliative needs of the population is key to reducing prolonged suffering related to the burden of living with life-limiting illness [5] and reduced quality of life [6]. In light of the growing international demand for palliative care [7] and the significant health and financial consequences associated with unmet palliative needs, health technologies have been explored as an opportunity to support palliative care delivery [3]. The utilization of health technologies to enable and support the provision of health services and the transmission of health information is referred to broadly as telehealth [3]. In this paper, the terms, “telehealth” and “health technologies” will be used interchangeably. Telehealth enables electronic modes of communication between patients and the care team (e.g. video visits, audio connections), and remote symptom monitoring and assessment [3]. Research demonstrates that telehealth can help improve symptom management and quality of life, decrease health care costs [8–11], and reduces risk of disease transmission due to decreasing direct contact [12].

A number of systematic reviews of research on the application of telemedicine in palliative care have been conducted [13–16]. Some studies demonstrated its feasibility [15, 16] and provided some evidence of its effectiveness for improving quality of care, documentation, cost, and communication in palliative care [13]. Others have focused on users’ information needs [13], patient-reported health outcomes [14], patient satisfaction, and health care use [15]. Health technology *acceptance*, however, has not been extensively explored within the context of palliative care specifically, despite the fact that studies have shown that acceptance acts as a key variable influencing technology adoption [17, 18]. Given the emergence of new health technologies and evolving attitudes as health systems adapt to rapidly changing circumstances, it is important to understand the acceptance of current technology applications in palliative care in order to gain insight into how they can be rapidly scaled up. Nuances between palliative care settings across various regions, and important differences between hospice and palliative care provision in a number of countries raise the need to further develop the existing knowledge base to explore technology acceptance in additional palliative care contexts. Compared to hospice care, which focuses on the palliation of patients who are in their remaining months of life, palliative care is distinct in its aim to improve the quality of life among patients with life-limiting or serious illnesses who are not necessarily close to death [19].

A limited number of studies have explored technology acceptance within palliative care. Some of the evaluated technologies allowed for monitoring and assessing symptoms [7, 18, 20, 21], while others for video consultations, exclusively [22]. Among these studies, factors supporting acceptance included demonstrated evidence that technology could enhance patient outcomes/experiences [20, 21] and could act as an adjunct to in-person care [7, 18]. Technologies that were tailored to meet the specific needs of different palliative care settings [22] also supported provider acceptance. Conversely, perceived lack of clinical relevance (of aspects of the technology) [20], lack of digital infrastructure to support technology [7, 18], and privacy concerns [22] were notable barriers to technology acceptance. Although health care providers were included in the palliative care studies previously mentioned, health administrator perspectives were excluded. Administrators are often closely involved in the procurement and/or coordination of health technologies and can potentially directly influence providers’ decision to use it on a regular basis. Therefore, health administrators’ perspectives are also important to consider to better understand technology acceptance in palliative care.

Our study sought to expand on previous research by providing a current and theoretically informed perspective on patients’ and providers’ acceptance of palliative care telehealth applications as viewed by health care providers and administrators in Ontario, Canada. We drew on the Technology Acceptance Model (TAM) [23] to explore the usefulness and ease of using health technologies for both providers and patients in the palliative care context. To our knowledge, this is the first Canadian study that focuses on acceptance of technologies in palliative care from the perspectives of both health administrators and providers.

## Methods

### Theoretical framework: technology acceptance model

The TAM centers on two belief constructs that have been found to significantly influence an individual’s acceptance of (intention to engage) a technology: 1) perceived usefulness; and 2) perceived ease of use [23]. This model contends that a strong relationship exists between one’s intention to use technology and their actual usage behavior [24]. Perceived usefulness is characterized by an individual’s belief that engaging a technology improves their job performance while perceived ease of use refers to their belief that using a technology requires minimal effort [23]. The TAM assumes that an individual’s perception of how easy a technology is to engage with impacts their evaluation of its usefulness [23]. Thus, we explored these key variables in our study.

### Study setting

We obtained ethical approval for the study from the Women’s College Hospital ethical review committee. Our study was a follow-up to two previous proof-of-concept (POC) implementations of patient- and provider-facing technologies in palliative care that were piloted by the Ontario Telemedicine Network (OTN) (a non-profit organization that facilitates virtually-enabled patient access to care, distance education, and remote meetings for health care providers and patients) in two regions in Ontario, Canada from September 2017 to March 2018. In one region, the piloted technology was a remote monitoring tool deployed as an adjunct to a home-based model of palliative care. The technology involved a set of survey instruments delivered on a tablet that was provided to and completed by patients or their caregiver(s) at home to capture routine information on their pain and symptom levels. Their data was then transmitted to a web-based database accessible by health administrators and providers to monitor patients for changes that potentially required either remote or in-person follow-up.

In the other region, the technology also supported a home-based model of palliative care. The technology consisted of a platform that acted as a central repository of information controlled by the patient and/or their caregiver(s). The technology supported self-management and could also act as a communication tool enabling video/audio touch-points between patients and the care team. The technology was designed as a personal electronic health record accessible to patients on their personal device. The personal electronic health record included goal-setting features, advance care and self-care planning, self-monitoring tools to track pain and symptoms, videoconferencing, and the potential to include other apps and features. The technology was intended to help support patients’ knowledge of their health status and engagement with their care.

While participants were recruited in the context of the two POC projects, participants were encouraged to consider their entire experience with the use of telehealth technologies in palliative care, even if this experience came from outside the POC projects. Some of the other technologies that participants spoke about included remote patient symptom assessment and monitoring tools, remote consultation tools, and digital decision support tools.

### Participant recruitment

Participants were recruited using purposive and snowball sampling whereby the study funder (OTN) recommended potential participants (*n* = 4 health administrators) who were previously involved in the pilot projects. We then expanded our recruitment by drawing on our professional networks and existing study participants to refer health providers and administrators via word-of-mouth (*n* = 14), including individuals outside the pilot projects who had relevant experience implementing/using various technologies in palliative care. Participants were contacted through telephone or email for (or to schedule) an introductory telephone call whereby the study objectives and details of participation were explained and to provide an opportunity to build rapport. Of the 23 individuals we contacted, two refused to participate and three dropped out due to lack of availability. In total, our study included 18 participants (seven health care providers [three physicians in palliative consultation teams/centers, two family physicians, two registered nurses] and 11 health administrators). Nine participants were involved to various extents in the pilot projects (seven health administrators, two health care providers) while the remaining nine included those with relevant telehealth experience in palliative care outside the pilot projects (four administrators, five health care providers).

### Data collection

We conducted 18 semi-structured, individual interviews with participants to explore their perceptions of the perceived usefulness and ease of using health technology in palliative care. Development of the interview guide was informed by the two belief constructs underlying the TAM (perceived usefulness and ease of use) as well as discussions with a family physician, a palliative care physician, and researchers with training and experience in qualitative methodologies and digital health evaluations. Questions centered on what supported the usefulness of health technologies (e.g. how technology could address current gaps in palliative care, positive impacts of using technology in palliative care, how technology could be integrated with workflow to optimize palliative care), and what improvements would be required to support their ease of utilization/adoption in palliative care (e.g. barriers or challenges that would need to be addressed). Semi-structured interview guides were used to provide a preliminary format for discussion, while enabling the exploration of common responses that emerged during interviews until data saturation was reached. Please see “Additional Files – Interview Guides”. Interviews were conducted by two female research assistants with masters and PhD training in qualitative methods and were conducted over the phone, lasting approximately 30 to 60 min. All interviews were audio-recorded and transcribed verbatim.

### Data analysis

The study scientific lead and two research assistants analyzed the data using inductive thematic analysis – a systematic approach in which the identification of meaningful themes (commonalities, patterns within the data) were derived from the data set [25]. We used (NVivo11) software to code (manage, organize, and categorize using descriptive labels) the data for subsequent analysis. In line with a theory-driven approach to thematic analysis [25], the interview data were analyzed in terms of the TAM and the overarching aim of the proposed research. Thus, analysis was oriented to exploring the usefulness of technologies and what supports their ease of use in palliative care. First, we identified (at random) and independently coded five transcripts and then compared codes as a group before coding the remaining transcripts. We identified passages within the dataset that corresponded to a common idea and assigned codes to group these portions of the data together. Using an iterative approach, we continually revisited and refined the codes throughout the analysis. We then reviewed the codes to generate themes based on our theoretical approach and research objective.

## Findings

Participants’ feedback centered on the usefulness of technology with respect to its capacity to support improved access to and integration of a palliative approach into standard care for individuals with life-limiting conditions. Responses also focused on key considerations for supporting the ease of using health technologies among patients and providers in palliative care. From our analysis, we identified four themes related to the usefulness and ease of engaging technologies in palliative care. Two themes related to perceived usefulness were: “enabling remote connection” and “information-sharing platform”, while “integration with existing IT systems” and “user-friendly with ready access to technical support” encompassed perceived ease of use.

### Perceived usefulness

#### Enabling remote connection

The potential for technology to facilitate timely access to palliative care was seen as a critical feature among participants. Specifically, the capacity for technology to quickly enable remote connections among patients, providers, and the care team was perceived as a potentially valuable means to circumvent some of the key challenges in palliative care. One participant who oversaw a palliative care program that used web-based portals to access patient information and telephone to communicate with patients shared their perception of how technology could potentially help support efficient and effective care:

*It just cuts down on the time, … travel and the aggravation. Some of our patients, they can’t go to an appointment so unless you can go to them … like I say, there’s one nurse after hours, covering the whole county for [one region], so the timeliness of an emergency visit is lacking in many instances, especially near death. Then we phone 911, and the ambulance takes them to the hospital, when there’s no really reason to go to the hospital. They’re not going to get any better care actually, and they might even get worse care at the hospital because they don’t have somebody on at the particular time, that is a palliative expert. I think the timeliness is just a huge benefit…* (Administrator)

Palliative care was viewed as a specialty that takes on patients at advanced stages of disease and was considered difficult to access because it tends to operate separately from other health services. Lack of integration of a palliative approach into standard care for individuals was perceived to result in inappropriate care for patients who required palliative services. Technology was seen as a possible way to help address the shortage and insufficient integration of palliative services into standard care by enabling patients to connect remotely with palliative care experts who would otherwise be difficult to access in a timely manner.

Participants perceived the capacity for technology to remotely connect patients and providers to be particularly valuable for patients who live considerable distances from the nearest palliative care provider and whose conditions often limit their ease of travel. Further, being able to easily connect with patients in a timely manner was considered especially important in the context of palliative care provision where routine symptom control is a key priority. An administrator who was involved in exploring digital decision support tools discussed their perception of how technology could be a potentially valuable solution for supporting timely symptom control by enabling regular, remote monitoring (e.g. tracking vitals, videoconferencing):

*Well, I think symptom control is always important. I’m just thinking about, number one, what worries people and, number two, what sends them to the ER. I think often what sends them to the ER is the lack of symptom control. … Often, we don’t know or it’s not quick enough, so they end up going to the ER before they’re even able to contact us. So, I think symptoms are always important.* (Administrator)

For participants, enabling remote connection meant improving access to palliative care in a way that supports optimal management of patients’ symptoms. One provider with experience using an asynchronous messaging tool to consult with specialists and patients discussed this in relation to the way in which technology could reconcile a drawback of in-person palliative care:

*So getting the optimal management of their symptoms and also means that the patients are not having to waste time in transportation on bumpy roads. There’s a lot of transferring on and off the stretcher and that kind of thing which can be quite uncomfortable for a patient who’s already in pain*. (Health Care Provider)

For participants, the usefulness of technology revolved around its ability to make remote patient-provider connections a possibility because *“really being available [and] accessible”* was viewed as a *“big part of palliative care”* (Administrator).

#### Information-sharing platform

Technology was seen as a potential way to help reconcile limited palliative care knowledge among primary providers by enabling remote consultation with palliative care specialists. One administrator discussed their perception of how telehealth had the potential to address service gaps in palliative care:

*…if there is a healthcare provider already in the home but maybe needing additional specialized palliative expertise then video conferencing can allow them to get that support in the immediate rather again them having to call somebody out to physically go to the home. It can stretch the amount of service that can be provided. It can enhance access to the service.* (Administrator)

This perspective was echoed by a provider who, in speaking about technology-supported palliative care explained that *“sharing information and communicating in networks will improve our ability to provide resources where resources are more limited”.* In this respect, technology was perceived as an information sharing platform that could support interdisciplinary collaboration and communication in a way that helps address limited palliative care expertise among providers. Indeed, in reflecting on their experience using an asynchronous provider-to-specialist communication tool for expert consult, one provider commented:

*As a specialist, I honestly really like it. I think that it is a good way to empower other specialists and to kind of … they ask a question and they get an answer. And then the next time they have a patient like that they say well this is what the answer was last time … so let’s try it this time. So kind of spreading knowledge … through asking some questions and getting some advice from someone with kind of a different perspective and a different level of training.* (Health Care Provider)

The information-sharing aspect of technology-supported palliative care delivery was considered advantageous for facilitating team communication in a way that supports coordinated and collaborative care among the interdisciplinary team. A provider participant who had previous experience using asynchronous messaging (i.e. email and text-messaging) to consult with patients and other providers, discussed the potential value of a telehealth communication tool that they were prospectively implementing:

*I think that the value would be to keep everyone on the same page, and make sure that we’re all communicating the plan to each other. Often, I arrive at a visit, and I don’t know what the plan is from the perspective of oncology, or I don’t know what the family doctor is doing. I think it just allows a more collaborative approach to care, where everyone’s roles are more clearly delineated.* (Health Care Provider)

By providing a platform that enables the care team to communicate and remain on the same page, technology could act as a mechanism for reconciling the fragmentation between the various providers involved in the care of patients with palliative needs.

### Perceived ease of use

#### Integration with existing IT systems

Participants highlighted the importance of ensuring that a new health technology can seamlessly integrate with existing technology systems in palliative care in order to support its ease of use. This was particularly important for providers who identified the potential burden of introducing a new technology as an add-on to already time-consuming documentation/charting practices in palliative care. Their attitudes toward and intention of using technology were strongly influenced by its capacity to integrate with existing health information systems to enhance workflow and productivity without imposing additional work. One provider who used an electronic medical record (electronic medical record) and asynchronous messaging (email and text-messaging) to communicate with patients and the health care team explained:

*If it’s an extra thing to do, then I won’t do it. It needs to be linked in with my EMR or with my email, I don’t want to add another platform to my life. That’s for me … for me to really adopt it, it would need to be easy, and ‘integrateable’ into my current technology use.* (Health Care Provider)

Many participants commented on the significant value of adapting new technologies in

a way that enables real-time communication with other existing health information technologies in practice (e.g. the electronic medical record [EMR]) such that information auto-populates across systems. One provider participant with experiencing using the technology commented on its lack of integration with other digital platforms in practice:

*But then you also find in healthcare there’s different programs for everything … If somehow that technology could be integrated into electronic nurses notes, that would be even better. If it would just show up in an assessments’ tab, then the nurses would have it in real time and be able to address it.* (Health Care Provider)

#### User-friendly with ready access to technical support

Participants’ discussions of the particular challenges that patients with palliative needs face suggest that it is important to ensure that technologies in palliative care are user-friendly. Participants felt that user-friendliness was important to prevent imposing “*additional cognitive burden*” when patients are in “*a stage in life when their capacity is fairly diminished, and there are multiple demands on them*” (Health Care Provider). The need to ensure user-friendliness and to provide patients with support in utilizing technology was especially apparent in participants’ perceptions regarding limited technological literacy among this patient population, with individuals who are often elderly and unfamiliar with technology. As one participant with experience using a remote patient symptom monitoring tool commented:

*I think if you are providing patients with technology that they’re not familiar with, it takes a long time for this patient population, I guess they’re mainly a 70-plus population, it takes them a long time to adapt to using this, really. Things that you think would be intuitive are not – so establishing a good baseline understanding for them, and practicing I think is super important to the success of it.* (Administrator)

Participants felt that patients’ hesitance to engage technology was rooted in their lack of knowledge in how to use it. Technologies that appear complicated were perceived to intimidate patients and subsequently prevent their engagement. In discussing the utility of the technology in one of the POC projects, one administrator who had experience using the tool commented:

*Yeah, I think it could be a little bit more user-friendly. The interface was attractive, I thought it was nice looking. But there are some pieces about it that was a little bit sophisticated, even looking at the user guide and the user manual. I really simplified that for people, I made a very simple version of it, knowing that some of the people were just going to start in very limited ways in using the [technology]. But when I looked at the user manual … I’m a caregiver myself, I have two boys with disabilities. When I looked at that, I thought, most caregivers like me, wouldn’t read through that, it just, it looked overwhelming. Yeah, I wouldn’t … I know if somebody handed that to me and said, good luck, I hope that you use it, I would just sit down and cry.* (Administrator)

This account suggests that placing the sole onus on patients to learn and engage with a technology could prevent them from using it. Their approach to simplifying the user manual to facilitate engagement demonstrates that it is imperative to provide patient users with support in utilizing technologies in palliative care. Additionally, direct and repeated exposure to technology with extensive support from providers such as home care nurses or personal support workers was seen as an important factor for fostering comfort with technology among those who have lower levels of technological familiarity*.* An administrator participant involved in implementing the OTN pilot project noted that, “*… the more and more we have been introducing [the technology], the more people are open to it”.*

Participants also highlighted the need to ensure that information housed in new technologies are easily accessible to the appropriate end-users, such as the patient’s circle of care. Participants were aware that concerns about privacy and confidentiality often act as a barrier to engagement among health care providers in particular. Indeed, they highlighted the impracticality of building safeguards into the technology to the extent that providers might find it challenging to access patient information in the platform. In reflecting on a previous experience of implementing an EMR into clinical practice and drawing on principles of implementation science, one provider perceived that:

*… in terms of how healthcare provider access these systems, there should be a digital identification that gives you certain permissions to access the parts that you need of the patient’s information and platform.* (Health Care Provider)

This provider emphasized the need to protect patients’ privacy without being “*paralytic*” through the development of a “*rational digital infrastructure”* that safeguards patient information while still enabling providers to access what they need on the technology with ease. Participants found that the inability to access patient information on the platform created additional burden (for both patients and providers) because it imposed the need to conduct repeat health assessments.

## Discussion

This study explored acceptance of telehealth among providers and administrators involved in palliative care. Acceptance depended on the potential to address major challenges in the field without imposing significant burden to both providers and patients. These challenges include insufficient availability of palliative care services; lack of integration of a palliative approach into the care of individuals with life-limiting conditions; and limited palliative care expertise among non-specialist providers caring for individuals with palliative needs [1].

### Perceived usefulness: improving access to palliative care and supporting interdisciplinary knowledge building and collaboration

In this study, the perceived usefulness of technology-supported palliative care was grounded in participants’ beliefs about the extent to which remote connection could improve access to care and optimize quality of life through better management of symptoms. Echoing previous research [7, 11, 26], participants in this study highlighted the value in being able to overcome time and geographical barriers to access care via technology (e.g. video visits, remote symptom monitoring). Participants emphasized that patients with palliative needs often experience rapid evolution of symptoms and require regular touchpoints with care providers to achieve adequate symptom control. Technology could enable frequent monitoring through remote assessments and the timely palliation of symptoms by staff with appropriate skills. Consistent with previous research [18, 27], participants felt that patients travelling to in-person appointments often results in increased pain and discomfort due to limited mobility or function. As evidenced by other research [3], participants highlighted the capacity for technology to support patients’ common desire to remain in the comfort of their own homes while receiving palliative care.

The value that participants placed on enabling connection suggests that technology can help foster a sense of human presence even in the absence of physical touch when it is used to creatively augment rather than replace a sense of connection with the care team. This perspective may help to address a common concern that technology could compromise human connection [28]. Because many patients with palliative needs still value face-to-face connection with their providers [7], it is critical to further explore when applications of technology would or would not be appropriate to help providers navigate the balance of technology-supported palliative care. Our findings suggest that telehealth could be an important channel for supporting feelings of connectedness among patients who would otherwise have limited access to palliative services, which is often in the case in low- and middle-income areas and even in developed countries [5, 29].

Participants also felt that technology could extend existing palliative care resources through information-sharing and capacity-building within the provider team. Participants reported that technology could be used to support knowledge sharing and collaboration between palliative experts and primary providers to support integrated models of care that incorporate a palliative approach. Thus, previous research suggesting the use of technology as a peer-support tool between palliative care specialists [7] could be expanded to include primary providers as they often lack deep palliative expertise. This might help to address a major health services gap [5] and support coordination of care considering that palliative care is predominantly provided by teams of various primary providers (e.g. physicians, nurses, personal support workers) that work in “shared care arrangements” with palliative specialists in many areas [3].

### Ease of use: improving Telehealth integration with existing IT systems and user-friendliness with ready access to technical support

Participants expressed a strong desire for telehealth to seamlessly integrate with existing health information systems to enhance job performance without being obtrusive. Our findings revealed the importance of “passive” technology integration; it was crucial for users to feel as though the technology was working *for* them as opposed to *against* them in ways that stunted productivity and impeded workflow. Interoperability, secure data transfer, and data synchronization between different technological platforms were seen as important measures for promoting efficiency, continuity of care, and increasing the likelihood of sustained technology adoption. Echoing previous research on health technology adoption [30–32], participants shared a common concern that implementing new technologies in palliative care would increase workload even if such innovations supported job performance. Participants’ perspectives support the theoretical assumption underlying the TAM that users’ intentions to engage a technology are heavily influenced by the perceived amount of effort it takes to utilize it [23]. Telehealth integration into existing IT systems has been shown to act as a key feature for supporting adoption of health technologies among providers [32–36]. This is particularly important in the palliative context where a vast and complex network of providers is often involved in a patient’s care [37] and coordination of care across independent organizations and health information systems is required. While concerns about protecting patients’ health information are often raised in health technology research [38], participants in this study discussed how such privacy measures could unintentionally impede ease of use if they are not thought through from a standpoint that balances confidentiality with practicality. They emphasized the need to design technological infrastructure to include rational digital permissions such that patients’ information is protected while also enabling providers to access what they require with reasonable ease. When providers find it challenging to find or access technological features/components, this acts as a barrier to technology engagement [39].

Consistent with previous research [40–42], participants felt that telehealth needs to be user-friendly and to include ready access to technical support. This may be especially critical for patients in palliative care who often face cognitive and physical limitations. The importance of tailoring health technologies to the specific needs of end-users in palliative care is consistent with findings from previous telehealth research in patients with dementia, who also experience capacity limitations [43]. In line with previous research [44] and a key assumption underlying the TAM [23] regarding ease of use, participants felt that repeated hands-on experiences with technology were important for fostering a sense of comfort, particularly among patients who were initially reluctant. Participants highlighted the value in providers demonstrating and condensing the user guide into a more digestible format to help support patient end-users’ ease of use. This would help address concerns raised in our study and elsewhere [7] with respect to the use of technologies among individuals who lack technological literacy, are elderly, or frail. Further research is needed to explore how technologies could be adapted to address the user needs of those with severe limitations in cognitive function, mobility, vision, and hearing.

### Limitations

While the interviews were conducted with individuals who had experience and/or knowledge of the piloted technologies or other technologies in palliative care, their level of exposure to these technologies varied. As a result, some participants were not able to provide in-depth feedback on certain technological aspects. However, our inclusion of participants with diverse experiences of telehealth in palliative care supports the breadth of our exploration and mirrors the variable exposure that technology users would have in the real world. Since recruitment was reliant on project leads within each region, we were not able to contact potential participants directly and were given few names of appropriate end-users of the technology. Moreover, while administrators were able to provide valuable insight into telehealth applications in palliative care, aspects of their feedback with respect to specific clinical features within technologies were limited due to their lack of involvement in direct patient care. Nevertheless, understanding health administrator perceptions of the usefulness and ease of using technology in palliative care is important since they are involved in procurement and coordination, and can inform future implementation of technologies in this context.

## Conclusion

Our findings lend support to previous research on technology utilization in palliative care with respect to its potential to support improved access to and quality of palliative care. Participants’ orientation to broad challenges in the field provided valuable insight that could help inform health technology implementations supporting palliative care in many jurisdictions that are rapidly needed as health systems are overwhelmed by pandemic response. Our results demonstrate that technologies designed to address the particular challenges of palliative care delivery (e.g. poor patient access to palliative care and insufficient knowledge of palliative care) are viewed as most useful among providers and administrators. However, the ease of using such technologies influences users’ intention to adopt it. Further, participants’ strong consideration of patients’ needs warrants further research exploring the key factors influencing patients’ acceptance of these technologies in palliative care and how such technologies could effectively interface with both clinical and patient end-users. While the needs and conditions of palliative care delivery will undoubtedly vary from region to region, the findings from this study provide a key starting-point to inform the implementation of digital tools to support the expansion of palliative care as acute care demand for health services rises due to pandemic response. This will hopefully continue to establish as an ongoing practice in health systems in the future.

## Supplementary information

**Additional file 1.** Interview Guides. Interview Guides for Health Care Administrators and Health Care Providers. Three interview guides for participant groups – health care administrators and health care providers within and outside of POC implementation projects.

## Data Availability

Interview transcripts cannot be made available as we did not receive consent from participants for sharing entire interview transcripts. Entire interview transcripts could potentially make it easier to identify these individuals, thus raising ethical concerns.

## Abbreviations

TAM: Technology Acceptance Model
OTN: Ontario Telemedicine Network
