# Supplementary material for: Using the technology acceptance model to explore health provider and administrator perceptions of the usefulness and ease of using technology in palliative care
Source: BMC Palliat Care. 2020 Sep 7;19:138. doi: 10.1186/s12904-020-00644-8 (PMC7476427; doi:10.1186/s12904-020-00644-8)
Supplement: Supplementary file 1 — Additional file 1. Interview Guides. Interview Guides for Health Care Administrators and Health Care Providers. Three interview guides for participant groups – health care administrators and health care providers within and outside of POC implementation projects. [file 12904_2020_644_MOESM1_ESM.docx]

**Health Care Provider Interview Guide - Within POC**

Intended for health care providers involved in the Telepalliative implementation projects

**Typical care model**

1. Please describe your current role within palliative care and within the Telepalliative program (if any).
2. Who comprises the patient’s circle of care and how does the team work together to deliver care?
   1. Probe: What types of HCPs comprise the team?
   2. What are their roles and how do those roles fit in with one another?
3. How do you currently communicate with other members of the care team, patients, and caregivers?
4. From your perspective, what are the major gaps in palliative care and how can technology be used to address them?
   1. Probe: Specific features to include and where efforts should be prioritized?
5. As the provider, what are your most significant/pressing needs? Do you think technology can address some of these needs? If so, how?

**Telepalliative care model**

1. How did the Telepalliative program influence your delivery of patient care?
   1. Probes: Advantages/disadvantages? (e.g. earlier identification of patients, length of time it took to document, ease of sharing info).
   2. How did the Telepalliative program influence your ability to care for the patient? (e.g. communication, quality of care/interactions, awareness of patient’s condition).
2. How could a Telepalliative model best be integrated into the clinical care model to support the delivery of care to palliative patients?
   1. Probes: What changes to the clinical care model are required for a Telepalliative model to be successful?
   2. Who should be the lead in implementing/sustaining these changes on a practice level?
   3. How does the model fit within pre-existing workflows?
   4. Whose buy-in do you need for implementation and how would you achieve this?
   5. How can this integrated model be sustained over time?
3. What is required for the Telepalliative program to improve continuity and quality of care compared to the standard model?
   1. Probes: What specific features are necessary?
   2. How could the technology be improved to enhance continuity of care?

**Health Care Provider Interview Guide - Outside of POC**

Intended for health care providers who were not involved in the Telepalliative implementation projects

1. From your perspective, what are the major gaps in palliative care?
2. Do you think technology can be used to address some of these gaps and if so, how?
   1. Probe: What specific features would be important to include in the technology and where should efforts be prioritized?
3. Do you use any type of virtual care in your practice? (e.g. videoconferencing, remote monitoring, e-mail, etc.).
   1. If yes: Could you tell me more about the type of virtual care you use in your practice with respect to its function, features, and how it fits into your workflow?
4. How could technology fit in with your workflow to optimize the palliative care you deliver?
   1. Probe: What could make its integration easier?
5. What are some potential positive and negative impacts of using technology in palliative care?
   1. Probe: What is the potential impact on patients, caregivers, providers, and the health care system?
6. What kind of patient populations do you think would be most suitable for a virtual care approach and why?
7. What are some barriers to using technology among patients and their caregivers? What are some barriers specific to providers?
   1. Probes: What are some administrative barriers?
   2. What are some barriers/challenges that may occur with respect to implementing a new technology in palliative care?
8. In your opinion, what would facilitate the use of technology among patients/caregivers/ providers?
   1. Probe: What would need to happen to implement a technological solution in palliative care (or in your practice)?
9. Who should lead the implementation and sustainment of technology in palliative care?

**Health Care Administrator Interview Guide**

Intended for people involved in the administrative aspects of the Telepalliative implementation projects

1. Can you tell me what your current role is within palliative care and what is your connection (if any) with any of the Telepalliative programs?
2. From your perspective, what are the major challenges in palliative care and how can technology be used to address them?
   1. Probes: Examples of areas are Patient Monitoring, Predictive Analytics, Decision Support, Communication and Care Coordination
   2. If you were to develop a technology for palliative care, what would it look like (e.g. features) and who would you develop it for (e.g. providers, patients, caregivers)?
3. What are your thoughts on the tele-palliative care model?
   1. Probe: Advantages, disadvantages?
   2. Specific features of the technology that could be improved?
   3. What features could be added/eliminated?
4. What are some of the challenges you perceive in implementing Telepalliative programs?
   1. Probes:
      1. Cost
      2. Quality
      3. Continuity
      4. Access
      5. Equity
      6. Change Management
5. What stakeholder groups are important to enable the success of a Telepalliative program and how would you engage them?
   1. Probe: E.g., for programs targeting cancer patients, it may be important to engage cancer organizations and oncology providers.
6. If you could do one thing to improve palliative care what would it be?
